# Geridonin and paclitaxel act synergistically to inhibit the proliferation of gastric cancer cells through ROS-mediated regulation of the PTEN/PI3K/Akt pathway

**DOI:** 10.18632/oncotarget.12166

**Published:** 2016-09-21

**Authors:** Sai-Qi Wang, Cong Wang, Li-Ming Chang, Kai-Rui Zhou, Jun-Wei Wang, Yu Ke, Dong-Xiao Yang, Hong-Ge Shi, Ran Wang, Xiao-Li Shi, Li-Ying Ma, Hong-Min Liu

**Affiliations:** ^1^ School of Pharmaceutical Sciences, Zhengzhou University, Zhengzhou, 450001, P.R. China

**Keywords:** gastric cancer, paclitaxel resistance, oridonin, synergism, PI3K/Akt pathway

## Abstract

Paclitaxel, a taxane, is a cytotoxic chemotherapeutic agent that targets microtubules. It has become a front-line therapy for a broad range of malignancies, including lung, breast, gastric, esophageal, and bladder carcinomas. Although paclitaxel can inhibit tumor development and improve survival, poor solubility, myelotoxicity, allergic reactions, and drug resistance have restricted its clinical application. Paclitaxel is frequently combined with other chemotherapeutics to enhance the antitumor effects and reduce side effects. We synthesized geridonin, a derivative of oridonin, and demonstrate that geridonin and paclitaxel act synergistically to inhibit the growth of gastric cancer cells. Importantly, geridonin enhanced the antitumor effects of paclitaxel without increasing toxicity *in vivo*. Mechanistic analysis revealed that administration of geridonin in combination with paclitaxel up-regulated the tumor suppressor PTEN and inhibited phosphorylation of Akt and MDM2. This led to the accumulation of p53 and induced apoptosis though the mitochondrial pathway. Thus, geridonin in combination with paclitaxel is a new treatment strategy for gastric cancer.

## INTRODUCTION

The taxanes (paclitaxel and docetaxel) are some of the most efficacious and broadly used chemotherapeutics worldwide [1]. Paclitaxel inhibits microtubule depolymerization, down-regulates Bcl-2 and Bcl-xL expression, and causes cell cycle arrest at the G2/M phase [2], thereby suppressing tumor growth [3]. Paclitaxel can improve survival and sensitize cancer cells to radiotherapy. Therefore, it is a first-line chemotherapy for many types of cancer including ovarian, breast, non-small cell lung, and esophageal [4]. However, the clinical applications of paclitaxel are limited due to side effects such as gastrointestinal reactions, allergies [5], cardiotoxicity, and drug resistance [6]. In order to improve the therapeutic efficacy and delay drug resistance, combination chemotherapies involving paclitaxel have been developed [7], but enhanced toxicity has been observed [8].

Oridonin is an ent-kaurene diterpenoid isolated from *Rabdosia rubescens.* It displays broad antitumor effects in various cancers including liver, breast, prostate, esophagus, stomach, and leukemia, and exhibits low toxicity to normal cells [9–12]. However, the clinical applications of oridonin are limited due to poor water solubility and unsatisfactory pharmacokinetic properties [13, 14]. As a consequence, oridonin derivatives have been synthesized, which are potential antineoplastic drugs [15–17].

Apoptosis-targeted therapies are effective because dysregulated cell death contributes to cancer development. Apoptosis is typically induced through death receptors (the extrinsic pathway) or the mitochondrial (intrinsic) pathway to eliminate damaged cells and maintain homeostasis [18]. In both pathways, active caspases 3 and 7 cleave poly (ADP-ribose) polymerase 1 (PARP1) in response to DNA damage [19].

Reactive oxygen species (ROS) including peroxides, superoxide, hydroxyl radical, and singlet oxygen, are vital chemical messengers in cell signaling and homeostasis [20]. They are produced as natural byproducts of normal oxygen metabolism and are balanced by a scavenging system. However, environmental stress (e.g. ultraviolet radiation and imbalances in the oxygen scavenging system) can contribute to an increase in ROS levels [20]. ROS regulate the mitochondrial pathway of apoptosis in both a direct and indirect manner. Hydrogen peroxide (H_2_O_2_) induces dimerization of Bax, which promotes translocation of Bax from the cytoplasm to the outer mitochondrial membrane. ROS can also indirectly mediate apoptosis through the p38 mitogen-activated protein kinase (MAPK), c-Jun N-terminal kinase (JNK), extracellular signal-regulated kinase (ERK), and Akt pathways [21].

The phosphoinositide 3-kinase (PI3K)/Akt signaling pathway regulates numerous cellular processes including cell proliferation, cell cycle progression, and apoptosis [22, 23]. Active PI3K mediates phosphorylation of Akt on Thr 308 in the catalytic domain and Ser 473 in the regulatory domain, which leads to partial and full activation of Akt, respectively [24]. Activated Akt controls various biological responses. It can suppress apoptosis by directly phosphorylating apoptotic signaling proteins or by modulating the activity of transcription factors [25].

**Figure 1:**
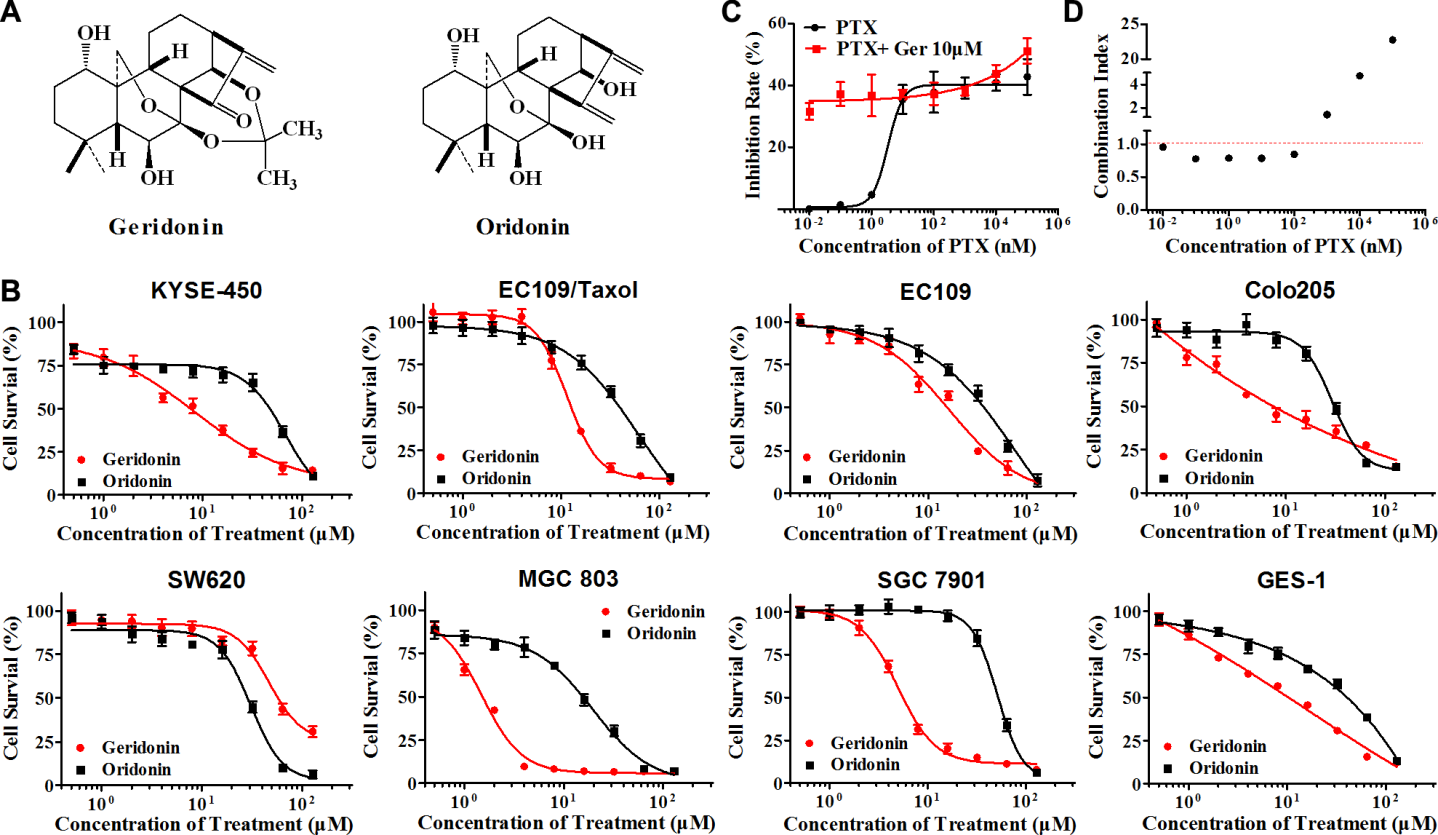
Geridonin (Ger) and paclitaxel (PTX) act synergistically to inhibit the proliferation of MGC 803 cells (**A**) Chemical structure of geridonin and oridonin. (**B**) Inhibitory effect of geridonin and oridonin treatment for 72 h. (**C**) Treatment with paclitaxel or paclitaxel in combination with 10 μM geridonin for 24 h inhibits the proliferation of MGC 803 cells. (**D**) CIs of paclitaxel and geridonin. MGC 803 cells were treated with geridonin, oridonin, paclitaxel, or geridonin plus paclitaxel for 24 h. Cell viability was assessed using MTT assays. The CIs were calculated using CompuSyn. Data are presented as the mean ± SD of triplicate tests.

In this study, we synthesized geridonin (Figure 1A and Supplementary Figure S1), a novel derivative of oridonin, and determined that it synergistically enhanced the anti-proliferation efficacy of paclitaxel against gastric cancer MGC 803 cells via ROS-induced inactivation of the PI3K/Akt pathway.

## RESULTS

### Geridonin synergistically enhances the paclitaxel-mediated growth inhibition of MGC 803 gastric cancer cells

Rabdosia rubescens has been used in China for the treatment of gastric and esophageal cancer [14]. We first evaluated the cytotoxicity of oridonin and geridonin (Figure 1A) on a series of human gastrointestinal cancer cell lines including esophageal (KYSE-450-, EC109, and EC109/Taxol [26]), colon (Colo205 and SW620), and gastric (MGC 803 and SGC 7901). All of the cell lines with the exception of SW620 showed higher sensitivity to geridonin than to oridonin (Figure 1B). Geridonin displayed a stronger growth-inhibitory effect against the paclitaxel-resistant cell line EC109/Taxol (derived from EC109) than against the parental cell line EC109. Both geridonin and oridonin were cytotoxic to GES-1 human gastric epithelial cells. The IC_50_ values of geridonin and oridonin at 72 h are shown in Table 1.

**Table 1:** IC_50_ values of geridonin and oridonin

Cell lines	IC_50_ (72 h, μM)	
	Geridonin	Oridonin
KYSE-450	7.15 ± 0.91	13.00 ± 1.72
EC109/Taxol	8.95 ± 1.11	33.53 ± 4.25
EC109	14.74 ± 3.02	29.71 ± 2.46
Colo205	9.72 ± 0.83	34.51 ± 3.77
SW620	69.28 ± 5.31	21.11 ± 1.93
MGC 803	1.60 ± 0.22	11.27 ± 1.53
SGC 7901	7.53 ± 1.02	26.64 ± 3.03
GES-1	10.16 ± 1.88	30.15 ± 2.47

To examine the combined effects of geridonin and paclitaxel, MGC 803 cells were treated with various concentrations of paclitaxel, either alone or in combination with 10 μM geridonin, for 24 h. Low doses of paclitaxel had a weak inhibitory effect on MGC 803 cell proliferation, while 10 μM geridonin significantly enhanced the anti-proliferative effect of paclitaxel (Figure 1C). We calculated the combination index (CI) using the CompuSyn software and the Chou-Talalay method, in which the combination index (CI) theorem offers a quantitative definition for an additive (CI = 1), synergistic (CI < 1), or antagonistic (CI > 1) effect of a drug combination. The CI values for 10 μM geridonin and paclitaxel (0.01–100 nM) were < 1, which was suggestive of synergism between geridonin and paclitaxel (Figure 1D). Therefore, we used 10 μM geridonin and 15 nM paclitaxel in all subsequent experiments.

**Figure 2:**
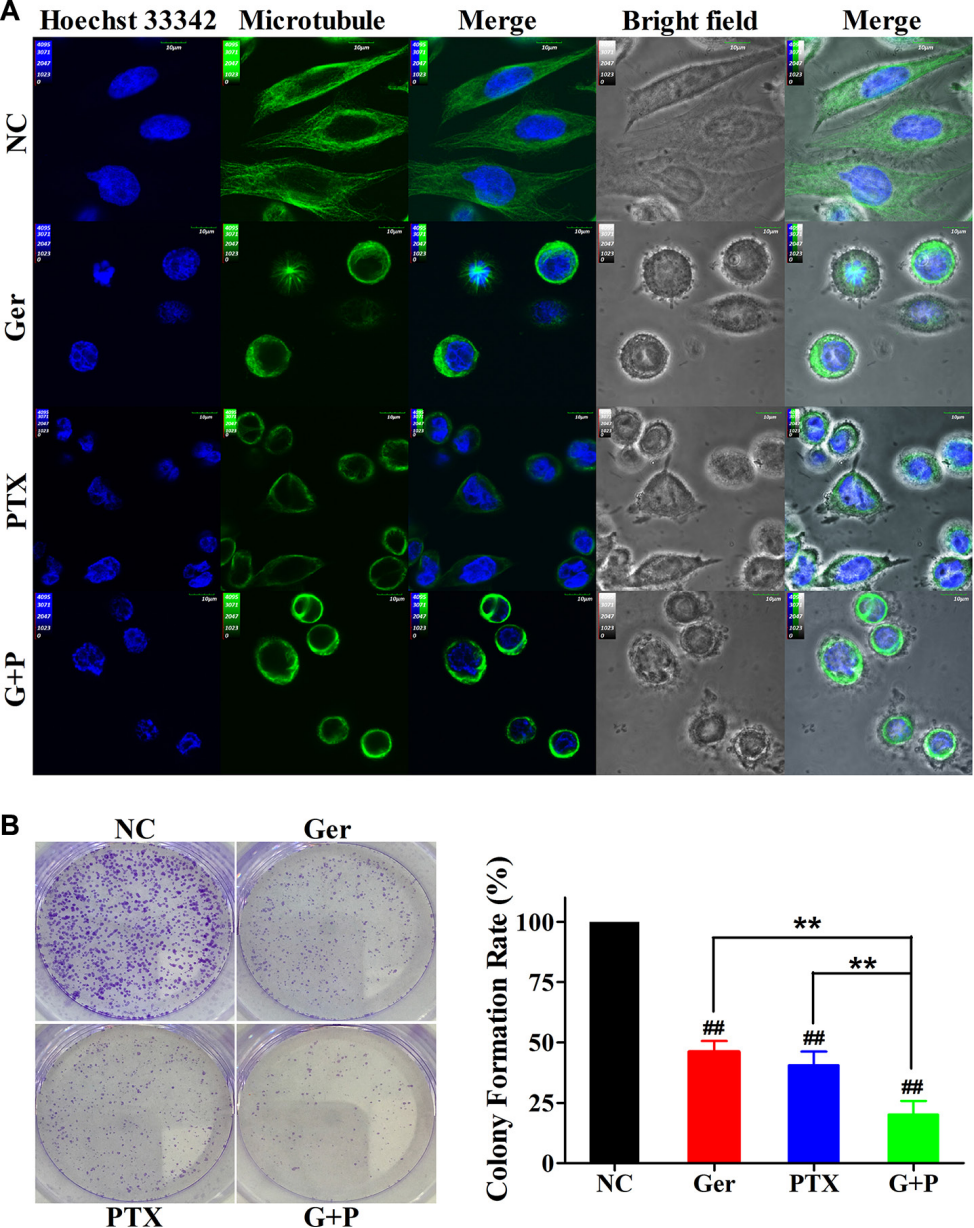
Geridonin enhances the inhibitory effects of paclitaxel on MGC 803 cells (**A**) Treatment of MGC 803 cells with geridonin (10 μM) and paclitaxel (15 nM) for 24 h perturbs microtubule organization. The α-tubulin staining is shown in green and the nuclei are stained with Hoechst 33342 (blue). Scale bar is 10 μm. (**B**) Treatment of MGC 803 cells with geridonin (10 μM) and paclitaxel (15 nM) for 12 h decreases colony formation. ^#^*P* < 0.05 versus the negative control; ^##^*P* < 0.01 versus the negative control; *P < 0.05; **P < 0.01.

Simultaneous treatment of MGC 803 cells with 10 μM geridonin and 15 nM paclitaxel for 24 h resulted in significant alterations in cell morphology (Figure 2A). Normal MGC 803 cells were spindle-shaped or polygonal with a homogeneous size distribution. Geridonin and paclitaxel treatment resulted in round-shaped cells. Cells treated with both geridonin and paclitaxel exhibited bubbling on the cell membrane (bright field). Normal MGC 803 cells had round nuclei. In contrast, nuclei in the treatment group had irregular shapes, were smaller, and in some cases exhibited perforation. Multinucleate cells were also observed by Hoechst 33342 staining. Since paclitaxel targets microtubules, we assessed changes in microtubule dynamics after treatment with geridonin and paclitaxel. In the untreated group, filamentous microtubules were clearly observed. In contrast, the majority of filamentous microtubules disappeared in the treatment group. In the geridonin and combination treatment groups, the remaining microtubules tightly embraced the nucleus, suggesting that geridonin and paclitaxel perturbed microtubule organization in MGC 803 cells.

We next performed colony formation assays to investigate the cytotoxicity of geridonin and paclitaxel [27]. MGC 803 cells were treated with either geridonin (10 μM), paclitaxel (15 nM), or geridonin plus paclitaxel for 12 h. The drugs were then removed and the MGC 803 cells cultured for an additional 10 days before the colonies were counted. Colony number and size decreased after treatment with geridonin or paclitaxel compared to the negative control (*P* < 0.01), and significantly fewer colonies were observed in the geridonin plus paclitaxel treatment group (*P* < 0.01 versus single-drug treatment) (Figure 2B).

### Geridonin enhances paclitaxel-induced apoptosis through activation of the caspase cascade

**Figure 3:**
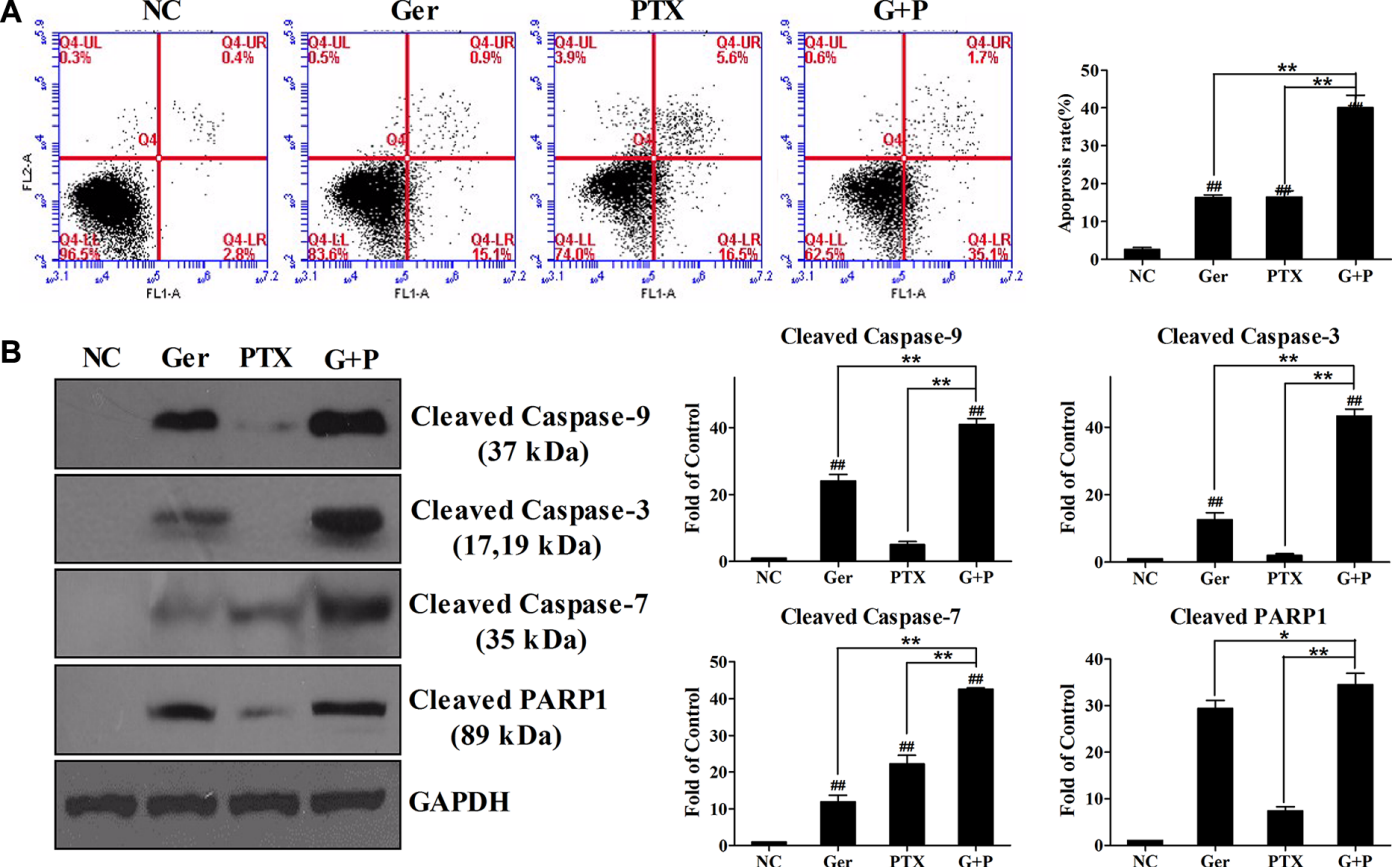
Geridonin enhances paclitaxel-induced apoptosis in MGC 803 cells (**A**) Apoptosis induced by treatment of MGC 803 cells with geridonin (10 μM), paclitaxel (15 nM), or geridonin plus paclitaxel for 24 h. (**B**) Activation of the caspase cascade after treatment of MGC 803 cells with geridonin (10 μM), paclitaxel (15 nM), or geridonin plus paclitaxel for 24 h. Data are presented as the mean ± SD of three independent experiments. ^#^*P* < 0.05 versus the negative control; ^##^*P* < 0.01 versus the negative control; *P < 0.05; **P < 0.01.

Apoptosis is frequently dysregulated during cancer progression. Therefore, induction of apoptosis has become an important strategy for cancer therapy [28, 29]. We examined apoptosis by flow cytometry to investigate the mechanisms underlying the synergistic effects of geridonin (10 μM) and paclitaxel (15 nM). The percentage of apoptotic cells after 24 h of treatment was 16.2% ± 1.16% and 16.4% ± 2.9% apoptosis (lower right quadrant), for the geridonin and paclitaxel-treated groups, respectively. The percentage of apoptotic cells was approximately 40.0% ± 5.9% after combination treatment for 24 h (Figure 3A). All of the treatment groups showed significant differences compared to the negative control group (P < 0.01, one-way ANOVA). Significant differences were also observed between the geridonin plus paclitaxel, and the geridonin and paclitaxel groups (*P* < 0.01, one-way ANOVA).

To analyze geridonin- and paclitaxel-induced apoptosis, we assessed caspase activation by western blotting. We found that 15 nM paclitaxel was not sufficient for activation of the caspase cascade. Treatment of the cells with 10 μM geridonin increased the levels of cleaved caspase-9, −3, −7, and PARP1 (Figure 3B). Combination treatment was more effective in activating caspase-9, −3, −7, and PARP1 than either agent alone, suggesting that geridonin may enhance the apoptosis-inducing effect of paclitaxel through the mitochondrial pathway.

### Geridonin and paclitaxel act synergistically to decrease the mitochondrial membrane potential in MGC 803 cells through modulating the expression of Bcl-2 family members

**Figure 4:**
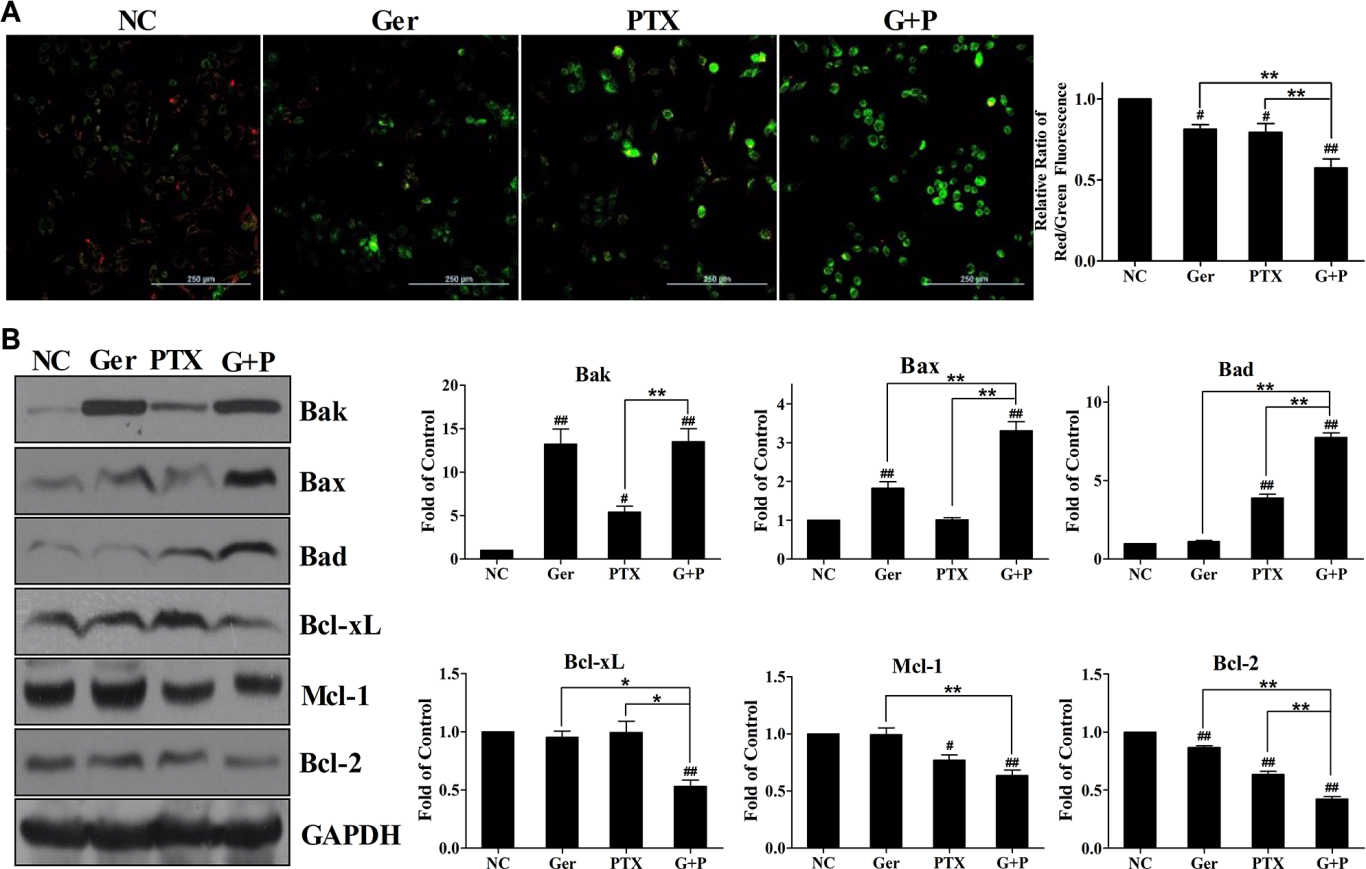
Geridonin exacerbates the paclitaxel-induced loss of mitochondrial membrane potential in MGC 803 cells (**A**) Measurement of the mitochondrial membrane potential of MGC 803 cells treated with geridonin (10 μM), paclitaxel (15 nM), or geridonin plus paclitaxel for 24 h. Scale bar is 250 μm. (**B**) Expression of Bcl-2 family members in MGC 803 cells following treatment with geridonin (10 μM), paclitaxel (15 nM), or geridonin plus paclitaxel for 24 h. GAPDH was used as a loading control. Data are presented as the mean ± SD of three independent experiments. ^#^*P* < 0.05 versus the negative control; ^##^*P* < 0.01 versus the negative control; *P < 0.05; **P < 0.01.

Apoptosis is regulated by the mitochondrial pathway and the death receptor pathway [30]. We measured the mitochondrial membrane potential in MGC 803 cells after geridonin, paclitaxel, or geridonin plus paclitaxel treatment (Figure 4A). Treatment of the cells with geridonin or paclitaxel for 24 h resulted in an 18.7% ± 5.0% (*P* < 0.05 versus the negative control) and 20.7% ± 9.5% (*P* < 0.05 versus the negative control) loss of mitochondrial membrane potential, respectively. Geridonin plus paclitaxel treatment resulted in a 42.7% ± 9.7% (*P* < 0.01 versus negative control; *P* < 0.01 versus each agent alone) loss of mitochondrial membrane potential. These data indicate geridonin and paclitaxel act synergistically to decrease the mitochondrial membrane potential in MGC 803 cells.

Members of the Bcl-2 family of proteins promote apoptosis in the mitochondrial pathway [30]. We found that geridonin primarily promoted the expression of the pro-apoptotic proteins Bak and Bax, while paclitaxel treatment resulted in up-regulation of Bad (Figure 4B) in MGC 803 cells. Geridonin plus paclitaxel treatment resulted in an increase in the levels of these pro-apoptotic proteins compared to control cells (*P* < 0.01). Geridonin and paclitaxel slightly altered the expression of Bcl-xL, Mcl-1, and Bcl-2. However, geridonin plus paclitaxel treatment reduced the levels of Bak and Bax (*P* < 0.01 versus negative control). In contrast, geridonin and oridonin did not affect the expression of Fas, DR4, DR5, or DcR1, which are all components of the death receptor pathway (Supplementary Figure S2). Overall, the data indicated that the synergetic effect of geridonin and paclitaxel on apoptosis was induced through the mitochondrial pathway through up-regulation of pro-apoptotic proteins and simultaneous down-regulation of anti-apoptotic members of the Bcl-2 family.

### Paclitaxel in combination with geridonin elevates intracellular ROS levels

**Figure 5:**
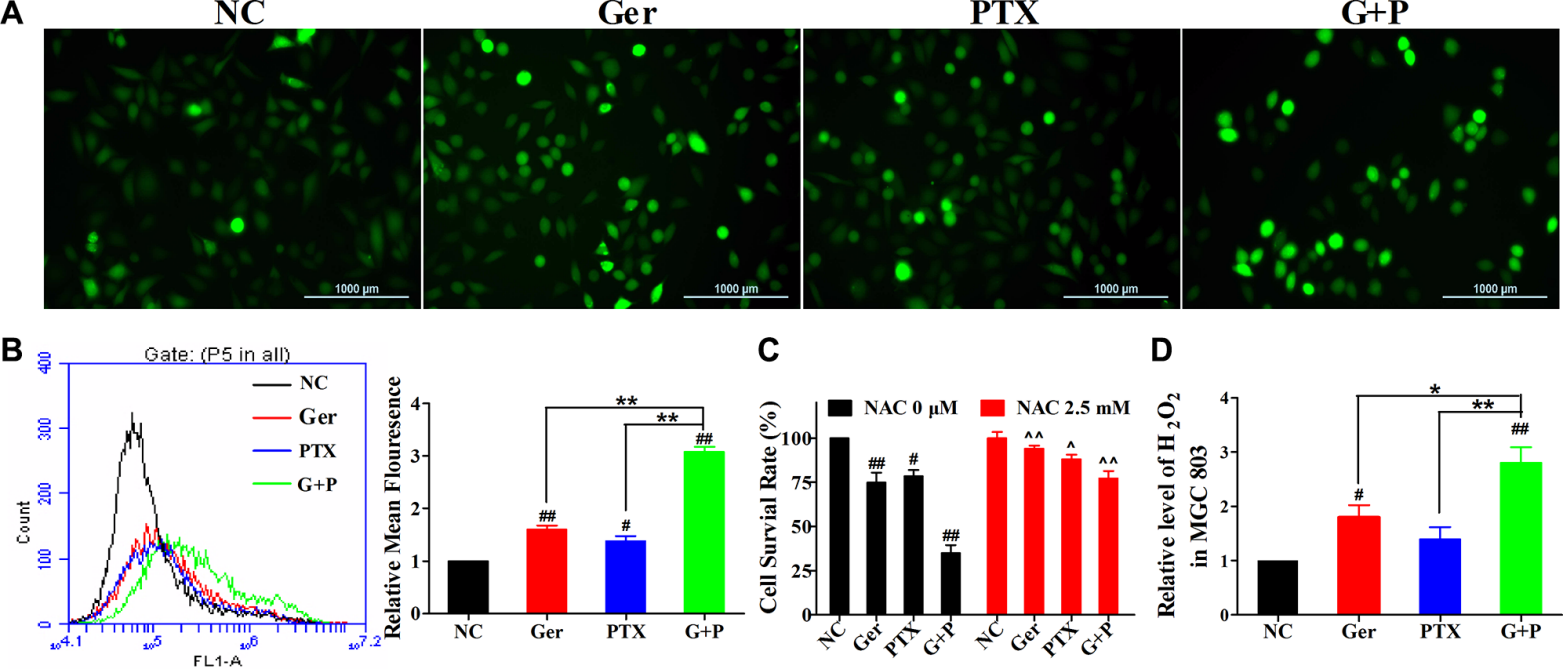
Geridonin and paclitaxel act synergistically to increase ROS levels in MGC 803 cells (**A**) ROS detection following treatment with geridonin (10 μM), paclitaxel (15 nM), or geridonin plus paclitaxel for 12 h. Scale bar is 1000 μm. (**B**) Quantitative analysis of intracellular ROS levels by flow cytometry. (**C**) Analysis of cell survival after treatment of MGC 803 treated with geridonin (10 μM), paclitaxel (15 nM), or geridonin plus paclitaxel in the presence or absence of NAC. Data are presented as the mean ± SD of three independent experiments. ^#^*P* < 0.05 versus the negative control; ^##^*P* < 0.01 versus the negative control; *P < 0.05; **P < 0.01; ^*P* < 0.05 versus the group that received the same treatment without NAC; ^^*P* < 0.01 versus the group that received the same treatment without NAC. (**D**) Levels of H_2_O_2_ in MGC 803 cells after treatment.

ROS regulate various cellular functions including proliferation, differentiation, and apoptosis. We evaluated intracellular ROS levels in MGC 803 cells treated with geridonin, paclitaxel, or geridonin plus paclitaxel for 12 h using the fluorescent probe dichloro-dihydro-fluorescein diacetate (DCFH-DA). Qualitative and quantitative analyses were performed using fluorescence microscopy (Figure 5A) and flow cytometry (Figure 5B), respectively. Treatment with 10 μM geridonin or 15 nM paclitaxel alone resulted in only a slight increase in ROS levels (1.59- and 1.38-fold compared to the negative control) (Figure 5B). Simultaneous treatment of MGC 803 cells with geridonin and paclitaxel increased ROS levels by 3.08-fold compared to the negative control (*P* < 0.01 versus the negative control and each drug alone). We next examined the effects of treating MGC 803 cells with the ROS scavenger N-acetylcysteine (NAC, 2.5 mM) in combination with geridonin, paclitaxel, or geridonin plus paclitaxel for 24 h. NAC reduced the anti-proliferative effects of geridonin (*P* < 0.01 versus the control), paclitaxel (*P* < 0.05), and geridonin plus paclitaxel (*P* < 0.01), suggesting that ROS may play a pivotal role in the synergism between geridonin and paclitaxel (Figure 5C). We also confirmed that geridonin and paclitaxel predominantly generated H_2_O_2_ in MGC 803 cells (Figure 5D).

### Geridonin and paclitaxel act synergistically through the PI3K/Akt pathway to inhibit the proliferation of MGC 803 cells

**Figure 6:**
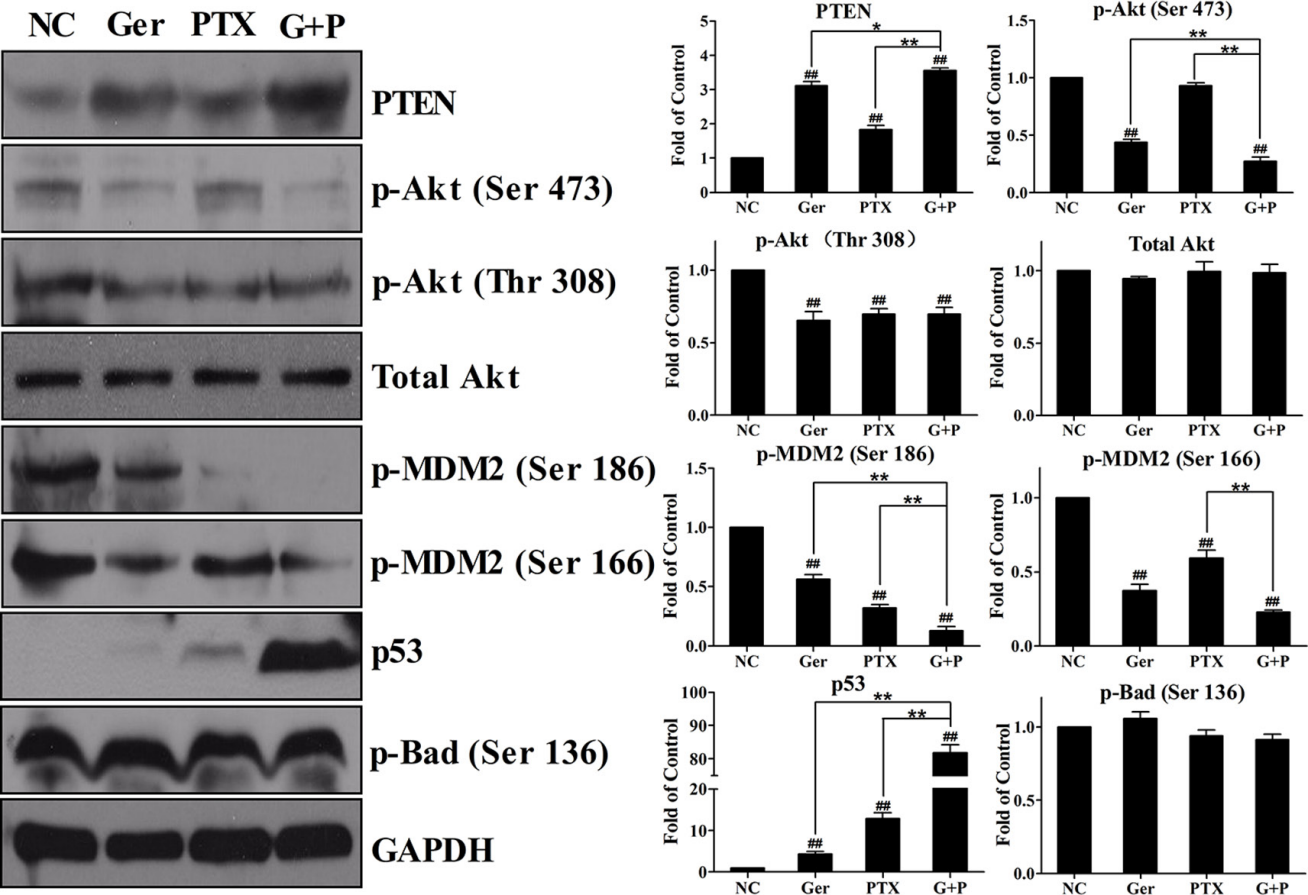
Geridonin and paclitaxel act synergistically to inhibit the PI3K/Akt pathway *in vitro* through regulation of PTEN expression Western blot analysis after treatment of MGC 803 cells with geridonin (10 μM), paclitaxel (15 nM), or geridonin plus paclitaxel for 24 h. GAPDH was used as a loading control. ^#^*P* < 0.05 versus the negative control; ^##^*P* < 0.01 versus the negative control; *P < 0.05; **P < 0.01.

Activated Akt inhibits apoptosis during cancer progression through several pathways and is an attractive therapeutic target [32]. Geridonin and paclitaxel inhibited Akt phosphorylation at both Thr 308 and Ser 473 (Figure 6). Geridonin plus paclitaxel treatment enhanced phosphorylation at Ser 473, but not at Thr 308, compared to treatment with either drug alone. Because Akt phosphorylation can be blocked by the tumor suppressor PTEN [31], we next measured PTEN expression. Geridonin and paclitaxel up-regulated PTEN expression to various extents, and combined treatment had a synergistic effect on PTEN expression. MDM2 is an important Akt substrate. Once phosphorylated by Akt, MDM2 can target the tumor suppressor p53 for degradation through ubiquitination. Phosphorylation of MDM2 at Ser 186 and Ser 166 residues was suppressed by geridonin, paclitaxel, and geridonin plus paclitaxel treatment, which resulted in inactivation of MDM2. Geridonin and paclitaxel also increased p53 expression, particularly when combined, which could have contributed to the deactivation of MDM2 (Figure 6). Akt also phosphorylates Bad at Ser 136 to block the antagonism between Bad and Bcl-2 or Bad and Bcl-xL and promote cell survival. However, none of the drugs altered phosphorylation of Bad at Ser 136 (Figure 6).

### Geridonin and paclitaxel act synergistically through the PI3K/Akt pathway to inhibit MGC 803 cell growth in a xenograft mouse model

**Figure 7:**
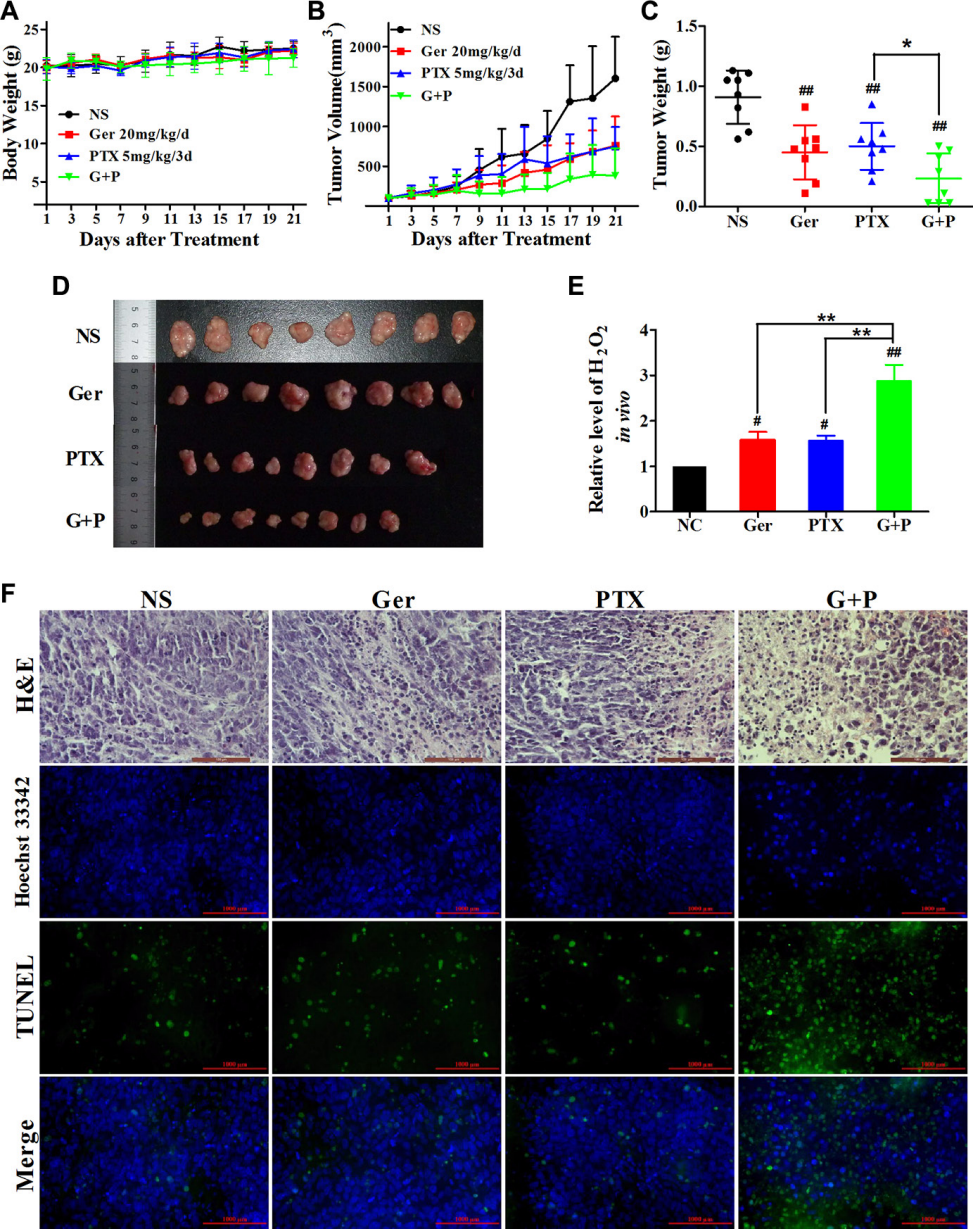
The antitumor effects of geridonin, paclitaxel, and geridonin plus paclitaxel *in vivo* (**A**) Body weight in athymic nude mice. MGC 803 cells were subcutaneously injected into the flanks of male athymic nude mice. Tumor volume was monitored for 3 weeks after treatment with geridonin, paclitaxel, or geridonin plus paclitaxel. (**B**) Tumor volume measurements for the athymic nude mice during treatment. (**C**) Tumor weight at the end of treatment (Day 21). (**D**) Tumors at the end of therapy (Day 21). (**E**) Levels of H_2_O_2_ in tumor tissue following treatment. (**F**) Representative hematoxylin and eosin staining (H&E) and TUNEL analysis of paraffin-embedded tumor tissue. Scale bar is 1000 μm. Data are presented as the mean ± SD, *n* = 8/group. ^##^*P* < 0.01 versus the negative control; *P < 0.05.

To analyze the synergism between geridonin and paclitaxel *in vivo*, we generated human tumor xenografts in nude mice. Both geridonin (20 mg/kg/d) and paclitaxel (5 mg/kg/3d) reduced tumor volume (Figure 7B) and weight (Figure 7C) compared to normal saline. After treatment for 21 days, the growth inhibition rates of geridonin and paclitaxel were 61.02% and 50.78% respectively. Slower tumor growth was observed in animals treated with geridonin plus paclitaxel compared to those treated with either drug alone. At the end of therapy, the inhibition rate of geridonin plus paclitaxel was 83.56% (*P* < 0.01 versus single-agent treatment). No obvious loss of body weight was observed in any of the four treatment groups, suggesting the therapies were well-tolerated (Figure 7A). Overall, the data indicated that geridonin enhanced the antitumor effects of paclitaxel without increasing toxicity. Additionally, we found that geridonin and oridonin generated a surplus of H_2_O_2_ in the tumor tissue (Figure 7E).

We next investigated the tumor-suppressive effects of geridonin, paclitaxel, geridonin plus paclitaxel using histological analysis and TUNEL assays. Hematoxylin and eosin staining revealed a high density of tumor cell nuclei in the normal saline-treated group that had a relatively uniform, spherical morphology. Treatment with either geridonin or paclitaxel alone resulted in the appearance of condensed nuclei that displayed darker blue staining compared to normal nuclei. The density of the nuclei also decreased, which was indicative of apoptosis. Following geridonin plus paclitaxel treatment, the majority of the cells exhibited condensed nuclei and a reduction in cytoplasmic and nuclear staining. These observations were indicative of apoptosis and necrosis (Figure 7F, row 1).

We performed TUNEL assays to detect DNA fragmentation, which results from apoptosis. Treatment with either geridonin or paclitaxel alone resulted in an increase in the green fluorescence signal in tumor tissues compared to the negative control, which was indicative of apoptosis (Figure 7F, rows 3 and 4). Treatment with paclitaxel plus geridonin enhanced apoptosis compared to either treatment alone.

**Figure 8:**
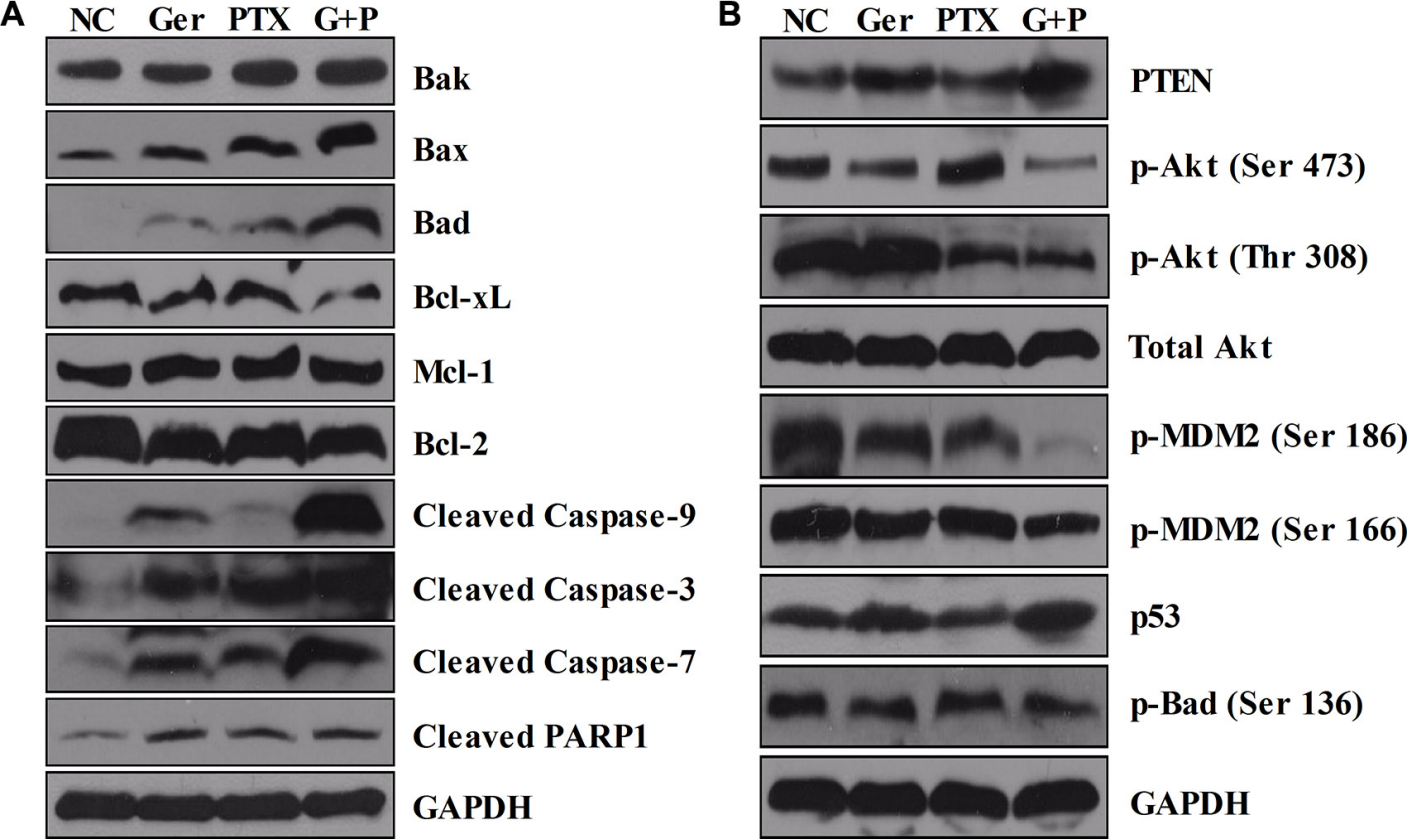
Western blot analysis of tumor tissue At the end of treatment, tumor tissue samples were lysed for western blot analysis. (**A**) Geridonin plus paclitaxel increases the expression of pro-apoptotic members of Bcl-2 family and decreases the levels of anti-apoptotic members resulting in activation of caspase −9, −3, −7, and PARP1 *in vivo*. (**B**) Geridonin and paclitaxel act synergistically to inhibit the PI3K/Akt pathway *in vivo* via up-regulation of PTEN expression. GAPDH was used as a loading control.

To further investigate the mechanisms underlying the synergistic anti-tumor effects of geridonin and paclitaxel *in vivo*, we analyzed tumor tissue by western blotting. Increased levels of PTEN and decreased phosphorylation of Akt both at Ser 473 and Thr 308 were observed *in vivo*, consistent with the synergist effects of geridonin and paclitaxel observed *in vitro*. Deactivation of Akt resulted in a reduction in phosphorylated MDM2 at Ser 186 and Ser 166, and accumulation of p53 (Figure 8B). As a consequence, pro-apoptotic Bcl-2 family members were up-regulated, while anti-apoptotic members were down-regulated. This resulted in activation of caspase-3, −9, −7, and PARP1 by geridonin, paclitaxel, or geridonin plus paclitaxel treatment (Figure 8A).

## DISCUSSION

To improve efficacy and reduce toxicity, chemotherapeutic drugs or other gene- [32], protein- or natural product-based [33] therapeutic agents are typically combined. In this study, we synthesized geridonin, which is a derivative of oridonin that exhibits enhanced cytotoxicity (Figure 1B). Geridonin enhanced the inhibitory effect of low dose paclitaxel on MGC 803 gastric cancer cell growth (Figure 1C) with CI values < 1 (Figure 1D). These results demonstrated a synergistic effect between geridonin and paclitaxel. A similar synergistic effect on cell growth was observed in colony formation assays (Figure 2B).

Paclitaxel targets β-tubulin to inhibit microtubule dynamics, and causes cell cycle arrest at the G2/M phase [34, 35]. Treatment of MGC 803 cells with paclitaxel resulted in alterations in cell morphology, the disappearance of normal microtubules, and multinucleate cells. Geridonin alone and in combination with paclitaxel induced similar changes in cell morphology and microtubule dynamics (Figure 2A). In addition to affecting microtubule dynamics, paclitaxel can induce apoptosis through activation or suppression of pro- and anti-apoptotic Bcl-2 family members [36–38]. We determined that geridonin enhanced paclitaxel-induced apoptosis (Figure 3A). Geridonin in combination with paclitaxel disrupted the balance between pro- and anti-apoptotic Bcl-2 family members resulting in destruction of the mitochondrial membrane and apoptosis mediated by the intrinsic pathway (Figure 4). Interestingly, geridonin and paclitaxel also suppressed the growth of MGC 803 xenograft tumors *in vivo*. Moreover, combination therapy was more effective in inhibiting tumor growth than either drug alone, and did not enhance the toxicity of paclitaxel. Histological analysis revealed that paclitaxel induced tumor cell apoptosis, which was enhanced by geridonin (Figure 7).

Redundant ROS induce apoptosis through both the extrinsic and intrinsic pathways [39]. We therefore examined the possibility that ROS induced apoptosis. ROS levels (e.g., H_2_O_2_) were elevated after treatment with either geridonin or paclitaxel alone, and combined treatment resulted in overproduction of H_2_O_2_ both *in vitro* and *in vivo* (Figure 5 and 7E).

Alterations in the PI3K/Akt pathway have been observed in many types of tumors [34]. This can promote cell survival and proliferation [40–42]. PTEN negatively regulates the Akt signaling pathway by dephosphorylating PIP3 to generate PIP2. The PTEN gene is frequently mutated in many different types of cancer [43, 44]. In this study, we observed up-regulation of PTEN after treatment with geridonin and paclitaxel alone, which was enhanced by geridonin plus paclitaxel treatment. PTEN degraded PIP3, which resulted in a decrease in Akt phosphorylation at Ser 473 and Thr 308. Inactivation of Akt decreased MDM2 phosphorylation, inhibited nuclear translocation, abolished MDM2-induced degradation of p53, and ultimately led to increased p53 expression and inhibition of tumor progression (Figure 8).

In summary, we have demonstrated that geridonin and paclitaxel act synergistically to suppress the growth of gastric cancer cells both *in vitro* and *in vivo* through ROS-mediated PTEN activation and deactivation of the PI3K/Akt signaling pathway. Therefore, geridonin in combination with paclitaxel may serve as a promising strategy for gastric cancer treatment.

## MATERIALS AND METHODS

### Drugs and antibodies

Geridonin was synthesized from oridonin in our laboratory. Paclitaxel was purchased from Meilun Biology Technology Co., Ltd. (Dalian, China). The Annexin V-FITC/propidium iodide (PI) Apoptosis Detection Kit was purchased from Jiamay Biotech Inc. (Beijing, China). The 3-(4, 5-Dimethylthiazol-2-yl)-2, 5-diphenyltetrazolium bromide (MTT), PI, and Hoechst 33342 were obtained from Beyotime Biotechnology (Shanghai, China). The primary antibodies against Bax, Bcl-2, and DcR1 were purchased from Abcam (Cambridge, MA, USA). The primary antibodies against cleaved caspase-9, −3, and −7, and cleaved PARP1, PTEN, phosphorylated Akt (Thr 308), phosphorylated Akt (Ser 473), and total Akt were from Cell Signaling Technology, Inc. (Danvers, MA, USA). The primary antibodies against Bak, Bad, Bcl-xL, Mcl-1, and Fas were purchased from EnoGene Biotechnology (Nanjing, China). The primary antibody against DR4 was purchased from Santa Cruz Biotechnology, Inc. (Dallas, TX, USA). The primary antibody against DR5 was purchased from Signalway Antibody (College Park, MD, USA). The primary antibody against GAPDH was obtained from Good Here Biotechnology Co., Ltd. (Hangzhou, China). Finally, the antibody against p53 was obtained from Santa Cruz Biotechnology, Inc.

### Cell culture

All cell lines were purchased from the Cell Bank of the Chinese Academy of Sciences (Shanghai, China). The MGC 803 cell line was authenticated by GENEWIZ Inc. (Suzhou, China). Colo205 and SW620 cells were cultured in DMEM (Solarbio, Beijing, China), and all other cell lines were cultured in RPMI 1640 (Solarbio, Beijing, China). Media was supplemented with 10% fetal bovine serum (Biological Industries, Kibbutz Beit Haemek, Israel), 100 units/mL penicillin, and 100 μg/mL streptomycin. The cells were cultured at 37°C in a 5% CO_2_ environment.

### Cell viability assays

Cell viability was evaluated using MTT assays [45]. The cells were seeded in 96-well plates at a density of 4,000 cells per well and then treated with geridonin, paclitaxel, or oridonin. After treatment with the indicated drugs, 20 μL of MTT was added to each well at a final concentration of 500 μg/mL. The plates were then incubated for 4 h at 37°C. The supernatant was removed after staining and 150 μL of dimethyl sulfoxide (DMSO) added to each well. The plates were vigorously shaken and the absorbance measured at 570 nm. The percent cytotoxicity was calculated using the following formula: % cytotoxicity = [1 – (absorbance of the experimental well - absorbance of the blank)/ (absorbance of the untreated control well - absorbance of the blank)] × 100. Drug interactions were analyzed using the median effect principle (Talalay-Chou method). The CI was calculated using the CompuSyn software, where a CI = 1 was indicative of an additive effect, a CI < 1 was indicative of a synergistic effect, and a CI > 1 was indicated of an antagonistic effect.

### Colony formation assays

A total of 1,000 MGC 803 cells per well were seeded in 6-well plates and treated with geridonin, paclitaxel, or geridonin plus paclitaxel for 12 h. Following the incubation, the medium was replaced and the cells cultured for an additional 10 days. Colonies were fixed with methanol, stained with crystal violet (Beyotime Biotechnology), and counted in ImageJ software (National Institutes of Health) [27, 46].

### Apoptosis assays

Apoptosis was analyzed using the Annexin V-FITC Apoptosis Detection Kit (KeyGEN BioTECH, Nanjing, China) according to the manufacturer's instructions. In brief, MGC 803 cells were washed twice with cold phosphate-buffered saline (PBS) and then resuspended in 500 μL binding buffer containing 5 μL Annexin V-FITC staining solution. The cells were incubated in the dark for 20 min, and then 5 μL PI staining solution was added to the cell suspension. After 5 min, apoptosis was detected using a BD Accuri^TM^ C6 flow cytometer (Becton Dickinson, Franklin Lakes, NJ, USA). The data were analyzed with the CFlow Plus software (Becton Dickinson).

### Immunofluorescence

MGC 803 cells were seeded on glass coverslips in 24-well plates (5 × 10^5^ cells per well) and incubated for 24 h. The cells were then treated with geridonin, paclitaxel, or geridonin plus paclitaxel for 24 h. Following the incubation, the cells were fixed with 4% (v/v) paraformaldehyde for 20 min at room temperature and then washed three times with PBS (5 min/wash). Next, the cells were permeabilized with 0.1% (v/v) Triton X-100 for 20 min at room temperature. After washing, the coverslips were blocked with 5% bovine serum albumin in PBS for 1 h at room temperature and then incubated with an α-tubulin primary antibody at 4°C overnight. After washing, coverslips were incubated with an Alexa Fluor® 488 Rabbit Anti-Goat IgG (H+L) for 1 h at room temperature. The cells were then washed and stained with Hoechst 33342 (5 μg/mL, Solarbio Life Sciences, Beijing, China) for 10 min. Images were collected using a laser scanning confocal microscope (Olympus, FV10i, Olympus Corporation, Tokyo, Japan).

### Mitochondrial membrane potential assays

The mitochondrial membrane potential was measured using JC-1 dye [45, 47]. MGC 803 cells were seeded on glass coverslips and treated with geridonin, paclitaxel, or geridonin plus paclitaxel for 24 h. MGC 803 cells were then stained with 5 μg/mL JC-1 (Beyotime Biotechnology) at 37°C for 20 min. Following the incubation, the cells were rinsed and the JC-1 signal visualized by confocal laser scanning microscopy (Nikon, A1, Tokyo, Japan). Monomers and J-aggregates were excited simultaneously with a 488 nm wavelength laser (green), while J-aggregates were excited using a 561 nm wavelength laser (red). Quantitative analysis (the relative ratio of red/green fluorescence) was performed using the NIS-Elements AR software (Nikon).

### ROS and H_2_O_2_ assays

ROS levels were probed using dichloro-dihydro-fluorescein diacetate (DCFH-DA) and detected by flow cytometry (BD Biosciences) and fluorescence microscopy (Nikon). H_2_O_2_ were measured using a Hydrogen Peroxide Assay Kit (Beyotime Biotechnology) [48] according to the manufacturer's instructions. After treatment for 12 h, 5 × 10^7^ cells were collected and lysed to measure H_2_O_2_ levels *in vitro*. Tumor tissue samples (20 mg) were lysed to measure H_2_O_2_ levels *in vivo*.

### Histological analysis and TUNEL assays

We prepared 5 μm tissue sections from paraffin-embedded tumor tissue samples. The sections were stained with hematoxylin and eosin for histological analysis. TUNEL assays of apoptosis were performed using the TUNEL Staining Kit (FITC dye; KeyGen Biotech, Nanjing, China). Apoptosis was visualized as green fluorescence. The cell nuclei were stained with Hoechst 33342 (5 μg/mL).

### *In vivo* assays of antitumor activity

All animal experiments were approved by the Ethics Committee of Zhengzhou University. Six-week-old male BALB/c nude mice (Silikejingda, Hunan, China) were housed in individually ventilated cages and maintained with food and sterile water. After approximately 1 week of acclimatization, MGC 803 cells (1.0 × 10^7^) were resuspended in 0.2 mL of saline and inoculated subcutaneously into the right forelimb of each mouse. When the tumor volume reached 100–150 mm^3^, the animals were randomized to the following treatment groups: NS (normal saline, 0.2 mL/kg/day, intravenous injection), paclitaxel (5 mg/kg/3 day, intravenous injection), geridonin (20 mg/kg/day, intravenous injection), and geridonin plus paclitaxel. Tumor length, width, and animal body weight were measured every 2 days. Tumor volume was calculated using the following formula: tumor volume (mm^3^) = length × width × width/2. Animals were sacrificed at the end of the experiment using the cervical dislocation method, and the tumors were excised and weighed. Tumors were stored at −80°C for later use in western blotting. Tumor samples were also fixed in 4% paraformaldehyde and embedded in paraffin for histological analysis.

### Western blotting

Cells or tumor tissue samples were lysed on ice for 30 min in RIPA buffer (Beyotime Biotechnology) containing 1% protease inhibitor cocktail and phosphatase inhibitor cocktail (Biotool, Houston, TX, USA). The lysates were then centrifuged at 12,000 *g* for 10 min at 4°C. The total protein concentrations were estimated using a BCA Protein Assay Kit (Solarbio Life Sciences, Beijing, China). Identical amounts of cell lysates were boiled for 10 min and then separated by SDS-PAGE. After electrophoresis, the proteins were transferred onto a nitro-cellulose membrane. The membrane was blocked with 5% non-fat milk in Tris-buffered saline and 0.05% Tween 20 (TBST) for 2 h at room temperature and then incubated with primary antibody overnight at 4°C. The membranes were washed three times with TBST and then incubated with horseradish peroxidase (HRP)-conjugated secondary antibodies (Zhongshan Golden Bridge, Beijing, China) for 2 h at room temperature. The membranes were then washed three times with TBST and immunoreactive bands visualized using enhanced chemiluminescence (ECL) reagent (Pierce Fast Western Blot Kit, Thermo Scientific, Waltham, MA, USA) according to the manufacturer's protocol. The relative expression ratios for the experimental and control groups were calculated based on density using the ImageJ software and the GAPDH signal as a reference.

### Statistical analysis

Each experiment was repeated at least three times. All data are presented as the mean ± standard deviation (SD) and were analyzed using the SPSS 17.0 software. Statistical significance was evaluated using one-way analysis of variance (ANOVA). Differences were considered to be statistically significant when *P* < 0.05.

## SUPPLEMENTARY MATERIALS FIGURES



## References

[1] Roy A, Bhattacharyya M, Ernsting MJ, May JP, Li SD (2014). Recent progress in the development of polysaccharide conjugates of docetaxel and paclitaxel. Wiley Interdiscip Rev Nanomed Nanobiotechnol.

[2] Pienta KJ (2001). Preclinical mechanisms of action of docetaxel and docetaxel combinations in prostate cancer. Semin Oncol.

[3] Fitzpatrick JM, de Wit R (2014). Taxane mechanisms of action: potential implications for treatment sequencing in metastatic castration-resistant prostate cancer. Eur Urol.

[4] Rowinsky EK (1997). The development and clinical utility of the taxane class of antimicrotubule chemotherapy agents. Annu Rev Med.

[5] Ozcelik B, Turkyilmaz C, Ozgun MT, Serin IS, Batukan C, Ozdamar S, Ozturk A (2010). Prevention of paclitaxel and cisplatin induced ovarian damage in rats by a gonadotropin-releasing hormone agonist. Fertil Steril.

[6] Barbuti AM, Chen ZS (2015). Paclitaxel Through the Ages of Anticancer Therapy: Exploring Its Role in Chemoresistance and Radiation Therapy. Cancers (Basel).

[7] Sonnenblick A, Eleyan F, Peretz T, Ospovat I, Merimsky O, Sella T, Peylan-Ramu N, Katz D (2015). Gemcitabine in combination with paclitaxel for advanced soft-tissue sarcomas. Mol Clin Oncol.

[8] Neijt JP, Engelholm SA, Tuxen MK, Sorensen PG, Hansen M, Sessa C, de Swart CA, Hirsch FR, Lund B, van Houwelingen HC (2000). Exploratory phase III study of paclitaxel and cisplatin versus paclitaxel and carboplatin in advanced ovarian cancer. J Clin Oncol.

[9] Kang N, Zhang JH, Qiu F, Chen S, Tashiro S, Onodera S, Ikejima T (2010). Induction of G(2)/M phase arrest and apoptosis by oridonin in human laryngeal carcinoma cells. J Nat Prod.

[10] Chen S, Gao J, Halicka HD, Huang X, Traganos F, Darzynkiewicz Z (2005). The cytostatic and cytotoxic effects of oridonin (Rubescenin), a diterpenoid from Rabdosia rubescens, on tumor cells of different lineage. Int J Oncol.

[11] Zhou GB, Kang H, Wang L, Gao L, Liu P, Xie J, Zhang FX, Weng XQ, Shen ZX, Chen J, Gu LJ, Yan M, Zhang DE (2007). Oridonin, a diterpenoid extracted from medicinal herbs, targets AML1-ETO fusion protein and shows potent antitumor activity with low adverse effects on t(8;21) leukemia *in vitro* and *in vivo*. Blood.

[12] Chen G, Wang K, Yang BY, Tang B, Chen JX, Hua ZC (2012). Synergistic antitumor activity of oridonin and arsenic trioxide on hepatocellular carcinoma cells. Int J Oncol.

[13] Xu J, Zhao JH, Liu Y, Feng NP, Zhang YT (2012). RGD-modified poly (D, L-lactic acid) nanoparticles enhance tumor targeting of oridonin. Int J Nanomedicine.

[14] Xu W, Sun J, Zhang TT, Ma B, Cui SM, Chen DW, He ZG (2006). Pharmacokinetic behaviors and oral bioavailability of oridonin in rat plasma. Acta Pharmacol Sin.

[15] Xu J, Yang J, Ran Q, Wang L, Liu J, Wang Z, Wu X, Hua W, Yuan S, Zhang L, Shen M, Ding Y (2008). Synthesis and biological evaluation of novel 1-O- and 14-O-derivatives of oridonin as potential anticancer drug candidates. Bioorg Med Chem Lett.

[16] Li D, Cai H, Jiang B, Liu G, Wang Y, Wang L, Yao H, Wu X, Sun Y, Xu J (2013). Synthesis of spirolactone-type diterpenoid derivatives from kaurene-type oridonin with improved antiproliferative effects and their apoptosis-inducing activity in human hepatoma Bel-7402 cells. Eur J Med Chem.

[17] Wang L, Li D, Xu S, Cai H, Yao H, Zhang Y, Jiang J, Xu J (2012). The conversion of oridonin to spirolactone-type or enmein-type diterpenoid: Synthesis and biological evaluation of ent-6,7-seco-oridonin derivatives as novel potential anticancer agents. Eur J Med Chem.

[18] Sola S, Morgado AL, Rodrigues CM (2013). Death receptors and mitochondria: two prime triggers of neural apoptosis and differentiation. Biochim Biophys Acta.

[19] Isabelle M, Moreel X, Gagne JP, Rouleau M, Ethier C, Gagne P, Hendzel MJ, Poirier GG (2010). Investigation of PARP-1, PARP-2, and PARG interactomes by affinity-purification mass spectrometry. Proteome Sci.

[20] Tong L, Chuang CC, Wu S, Zuo L (2015). Reactive oxygen species in redox cancer therapy. Cancer Lett.

[21] Wu CC, Bratton SB (2013). Regulation of the intrinsic apoptosis pathway by reactive oxygen species. Antioxid Redox Signal.

[22] Chang F, Lee JT, Navolanic PM, Steelman LS, Shelton JG, Blalock WL, Franklin RA, McCubrey JA (2003). Involvement of PI3K/Akt pathway in cell cycle progression, apoptosis, and neoplastic transformation: a target for cancer chemotherapy. Leukemia.

[23] Liang J, Slingerland JM (2003). Multiple roles of the PI3K/PKB (Akt) pathway in cell cycle progression. Cell Cycle.

[24] King D, Yeomanson D, Bryant HE (2015). PI3King the lock: targeting the PI3K/Akt/mTOR pathway as a novel therapeutic strategy in neuroblastoma. J Pediatr Hematol Oncol.

[25] Shaw RJ, Cantley LC (2006). Ras, PI (3)K and mTOR signalling controls tumour cell growth. Nature.

[26] Wang C, Guo LB, Ma JY, Li YM, Liu HM (2013). Establishment and characterization of a paclitaxelresistant human esophageal carcinoma cell line. Int J Oncol.

[27] Franken NAP, Rodermond HM, Stap J, Haveman J, van Bree C (2006). Clonogenic assay of cells *in vitro*. Nat Protoc.

[28] Koff JL, Ramachandiran S, Bernal-Mizrachi L (2015). A time to kill: targeting apoptosis in cancer. Int J Mol Sci.

[29] Fulda S (2015). Targeting extrinsic apoptosis in cancer: Challenges and opportunities. Semin Cell Dev Biol.

[30] Hardwick JM, Chen YB, Jonas EA (2012). Multipolar functions of BCL-2 proteins link energetics to apoptosis. Trends Cell Biol.

[31] Martelli AM, Evangelisti C, Chappell W, Abrams SL, Basecke J, Stivala F, Donia M, Fagone P, Nicoletti F, Libra M, Ruvolo V, Ruvolo P, Kempf CR (2011). Targeting the translational apparatus to improve leukemia therapy: roles of the PI3K/PTEN/Akt/mTOR pathway. Leukemia.

[32] Ediriwickrema A, Zhou J, Deng Y, Saltzman WM (2014). Multi-layered nanoparticles for combination gene and drug delivery to tumors. Biomaterials.

[33] Kim TH, Shin YJ, Won AJ, Lee BM, Choi WS, Jung JH, Chung HY, Kim HS (2014). Resveratrol enhances chemosensitivity of doxorubicin in multidrug-resistant human breast cancer cells via increased cellular influx of doxorubicin. Biochim Biophys Acta.

[34] Jordan MA, Toso RJ, Thrower D, Wilson L (1993). Mechanism of mitotic block and inhibition of cell proliferation by taxol at low concentrations. Proc Natl Acad Sci U S A.

[35] Long BH, Fairchild CR (1994). Paclitaxel inhibits progression of mitotic cells to G1 phase by interference with spindle formation without affecting other microtubule functions during anaphase and telephase. Cancer Res.

[36] Srivastava RK, Mi QS, Hardwick JM, Longo DL (1999). Deletion of the loop region of Bcl-2 completely blocks paclitaxel-induced apoptosis. Proc Natl Acad Sci USA.

[37] Strobel T, Kraeft SK, Chen LB, Cannistra SA (1998). BAX expression is associated with enhanced intracellular accumulation of paclitaxel: a novel role for BAX during chemotherapy-induced cell death. Cancer Res.

[38] Miller AV, Hicks MA, Nakajima W, Richardson AC, Windle JJ, Harada H (2013). Paclitaxel-induced apoptosis is BAK-dependent, but BAX and BIM-independent in breast tumor. Plos One.

[39] Ozben T (2007). Oxidative stress and apoptosis: impact on cancer therapy. J Pharm Sci.

[40] Peltier J, O'Neill A, Schaffer DV (2007). PI3K/Akt and CREB regulate adult neural hippocampal progenitor proliferation and differentiation. Dev Neurobiol.

[41] Chang F, Lee JT, Navolanic PM, Steelman LS, Shelton JG, Blalock WL, Franklin RA, McCubrey JA (2003). Involvement of PI3K/Akt pathway in cell cycle progression, apoptosis, and neoplastic transformation: a target for cancer chemotherapy. Leukemia.

[42] Petrulea MS, Plantinga TS, Smit JW, Georgescu CE, Netea-Maier RT (2015). PI3K/Akt/mTOR: A promising therapeutic target for non-medullary thyroid carcinoma. Cancer Treat Rev.

[43] Chen Z, Trotman LC, Shaffer D, Lin HK, Dotan ZA, Niki M, Koutcher JA, Scher HI, Ludwig T, Gerald W, Cordon-Cardo C, Pandolfi PP (2005). Crucial role of p53-dependent cellular senescence in suppression of Pten-deficient tumorigenesis. Nature.

[44] Liu JC, Wang DY, Egan SE, Zacksenhaus E (2016). Common and distinct features of mammary tumors driven by Pten-deletion or activating Pik3ca mutation. Oncotarget.

[45] Liao W, Zhang R, Dong C, Yu Z, Ren J (2016). Novel walnut peptide-selenium hybrids with enhanced anticancer synergism: facile synthesis and mechanistic investigation of anticancer activity. Int J Nanomedicine.

[46] Kawahara T, Inoue S, Ide H, Kashiwagi E, Ohtake S, Mizushima T, Li P, Li Y, Zheng Y, Uemura H, Netto GJ, Ishiguro H, Miyamoto H (2016). ZKSCAN3 promotes bladder cancer cell proliferation, migration, and invasion. Oncotarget.

[47] Liao W, Lai T, Chen L, Fu J, Sreenivasan ST, Yu Z, Ren J (2016). Synthesis and Characterization of a Walnut Peptides-Zinc Complex and Its Antiproliferative Activity against Human Breast Carcinoma Cells through the Induction of Apoptosis. J Agric Food Chem.

[48] Shi R, Hu C, Yuan Q, Yang T, Peng J, Li Y, Bai Y, Cao Z, Cheng G, Zhang G (2011). Involvement of vascular peroxidase 1 in angiotensin II-induced vascular smooth muscle cell proliferation. Cardiovasc Res.

