# Supplementary material for: Geridonin and paclitaxel act synergistically to inhibit the proliferation of gastric cancer cells through ROS-mediated regulation of the PTEN/PI3K/Akt pathway
Source: Oncotarget. 2016 Sep 21;7(45):72990–3002. doi: 10.18632/oncotarget.12166 (PMC5341958; doi:10.18632/oncotarget.12166)
Supplement: Supplementary file 1 [file oncotarget-07-72990-s001.pdf]

# Geridonin and paclitaxel act synergistically to inhibit the proliferation of gastric cancer cells through ROS-mediated regulation of the PTEN/PI3K/Akt pathway

## Supplementary Materials

## SUPPLEMENTARY DATA

### Synthesis of geridonin and preparation of the ligand solution

Oridonin (5.0 g) was dissolved in acetone (120 mL) and 2, 2-dimethoxypropane (20 mL) and catalytic p-toluenesulfonic acid (PTSA) added at room temperature. The reaction mixture was heated to 50°C and maintained for 1.5 h under argon. Dilute $NaHCO_3$ solution was added once the mixture had cooled to room temperature. The mixture was then extracted three times with $CHCl_3$. The combined organic layers were dried over anhydrous $Na_2SO_4$ and the solvent removed under vacuum. The residue was then recrystallized from methanol affording the title compound geridonin as the white needle solid (yield 88.3%) with a melting point of 219–221°C.

### Nuclear magnetic resonance (NMR) spectroscopy of geridonin

$^1$H NMR and $^{13}$C NMR spectra were recorded on a Bruker 400 and 100 MHz spectrometer (Billerica, MA, USA), respectively. $^1$H-NMR ($CDCl_3$) δppm: 6.14, 5.55 (each 1H, s, H2-17), 5.90 (1H, d, J = 11.0 Hz, C-6-OH), 4.79 (1H, s, H-14), 4.24, 4.04 (each 1H, d, J = 10.0Hz, H2-20), 3.87 (1H, dd, J = 10.4, 8.0 Hz, H-6), 3.46 (1H, m, H-1), 3.06 (1H, d, J = 9.2Hz, H-13), 2.52 (1H, dt, J = 8.8, 13.6Hz, H-12), 1.48-1.62 (2H, m, H-2), 1.65, 1.32 (each 3H, s, $CH_3 \times 2$), 1.15 (6H, s, $CH_3 \times 2$); $^{13}$C NMR ($CDCl_3$) δppm: 205.53 (C-15), 150.88 (C-16), 119.97 (C-17), 100.99 (C-21), 94.86 (C-7), 74.52 (C-14), 73.08 (C-20), 70.06 (C-1), 62.68 (C-8), 58.96 (C-9), 56.34 (C-11), 51.08 (C-5), 40.90 (C-12), 40.35 (C-13), 38.99 (C-10), 33.67 (C-3), 33.30 (C-18), 30.53 (C-4), 30.20 (C-3), 29.76 (C-22), 25.51 (C-23), 22.55 (C-6), 20.01 (C-2).

### High-resolution electrospray ionization mass spectrometry of geridonin

High-resolution electrospray ionization mass spectra were recorded on a Waters Micromass Q-Tof Micromass spectrometer (Milford, MA, USA). Calcd. $C_{23}H_{32}O_6$, $[M+Na]^+$ *m/z*: 427.2099, found: 427.2098.

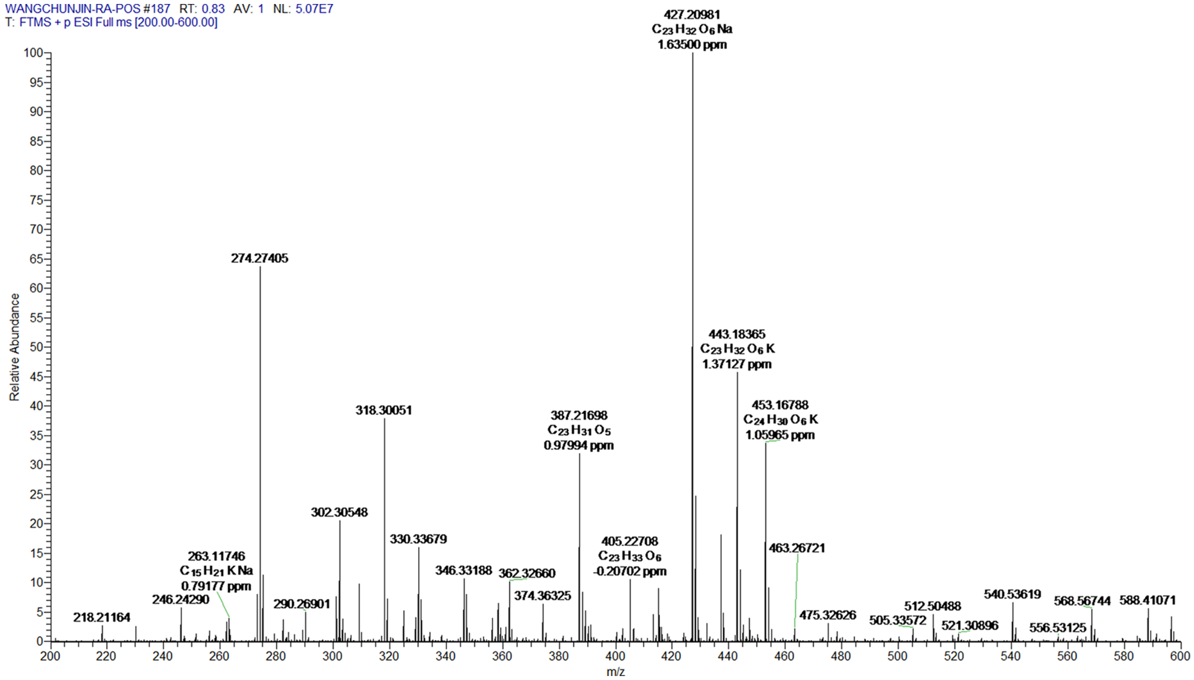


**Supplementary Figure S1: High resolution electrospray ionization mass spectrometry of geridonin.** High-resolution electrospray ionization mass spectra of geridonin were recorded on a Waters Micromass Q-Tof Micromass spectrometer.

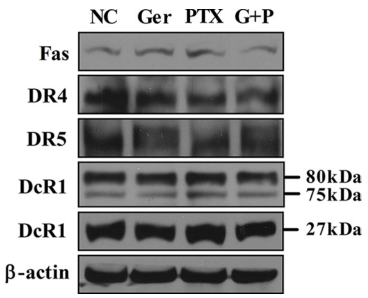


**Supplementary Figure S2: Geridonin and paclitaxel do not affect the expression of Fas, DR4, DR5, or DcR1.** Western blot analysis was performed after treatment with geridonin (10 μM), paclitaxel (15 nM), or geridonin plus paclitaxel for 24 h. β-actin was used as a loading control.
